# Manipulation of Rice Straw Silage Fermentation with Different Types of Lactic Acid Bacteria Inoculant Affects Rumen Microbial Fermentation Characteristics and Methane Production

**DOI:** 10.3390/vetsci8060100

**Published:** 2021-06-04

**Authors:** Ehsan Oskoueian, Mohammad Faseleh Jahromi, Saeid Jafari, Majid Shakeri, Hieu Huu Le, Mahdi Ebrahimi

**Affiliations:** 1 Department of Research and Development, Arka Biotechnology Corporation, Mashhad 1696700, Iran; mfjahromi@gmail.com; 2 Department of Veterinary Public Health, Faculty of Veterinary Science, Chulalongkorn University, Bangkok 10330, Thailand; jafarisaeid@rocketmail.com; 3 Department of Medicine, The University of Washington, Seattle, WA 98109, USA; mshakeri@uw.edu; 4 Faculty of Veterinary and Agricultural Sciences, The University of Melbourne, Parkville, VIC 3010, Australia; lhhieu.hua@gmail.com; 5 Department of Veterinary Preclinical Sciences, Faculty of Veterinary Medicine, University Putra Malaysia, Serdang 43400, Selangor, Malaysia; mehdiebrahimii@gmail.com

**Keywords:** in vitro, lactic acid bacteria, methane, microbial population, rice straw silage, rumen

## Abstract

Bacterial inoculants are known to improve the quality of silage. The objectives of the present study were to evaluate the effects of different types of lactic acid bacteria (LAB; *L. plantarum*, *L. salivarius, L. reuteri, L. brevi,* and *S. bovis*) inoculation (10^6^ cfu/ DM) on rice straw silage quality and to determine these effects on ruminal fermentation characteristics, digestibility and microbial populations in an in vitro condition. Inoculated rice straw was ensiled for 15 and 30 days. For the in vitro study, rumen fluid was obtained from three rumen-fistulated bulls fed on mixed forage and concentrate at 60:40 ratio twice daily. Inoculation with LAB improved *(p* < 0.05) the rice straw silage quality as indicated by higher dry matter and crude protein contents, decreased pH and butyric acid, and increased propionic acid and LAB numbers, especially after 30 days of ensiling. Results from the in vitro study revealed that starting with the addition of LAB to rice straw silage improved in vitro fermentation characteristics such as increased total volatile fatty acids and dry matter digestibility *(p* < 0.05). LAB treatments also decreased methane production and methane/total gas ratio after 15 and 30 days of ensiling. From the rumen microbial population perspective, cellulolytic, and fungal zoospores were enhanced, while protozoa and methanogens were decreased by the LAB treatments. Based on these results, it could be concluded that inoculating rice straw silage with LAB (especially for *L. plantarum* and *S. bovis*) improved silage quality, rumen fermentation parameters and microbial populations in vitro.

## 1. Introduction

The use of agricultural by-products is increasing because of limitations in food sources for livestock, which result in economic and environmental concerns. Rice straw, a major agricultural by-product, is routinely utilized as a feed source for ruminants in many regions of East and South-East Asia [1]. In Malaysia, rice straw is one of the most abundant agricultural by-products [2]. The production of rice straw in Malaysian fields has been estimated as 1,933,000 tons annually [3]. Rice straw surpluses are increasing due to regional restrictions and disposal by in-field burning (for example, in California, USA), and thus there is an even greater opportunity for using as a livestock feed if it is sufficiently nutritive. However, rice straw has very low nutritive values, with low crude protein content and metabolic energy for ruminants [3]. Technologies to create a high-quality animal feed from agricultural residues need to be developed. In ensiling, water-soluble carbohydrates are utilized by lactic acid bacteria (LAB) under anaerobic conditions to produce organic acids such as lactic acid, which results in reduced pH, inhibited growth of harmful bacteria and results in good quality silage [4]. Silage feeding is also a way to enhance livestock production, not only in the tropics but in temperate climates, especially during periods of inadequate supply of fresh forage. According to the literature, LAB (homofermentative and heterofermentative bacteria), which are widely used as inoculants, increase the concentration of lactic acid while reducing the pH and the concentration of ammonia nitrogen (NH_3_-N) in silage [5]. Several studies have shown the effectiveness of LAB on the feed quality of rice straw [6,7,8,9]. Besides, the studies mentioned that adding LAB increased the lactic acid content of silage, increased dry matter digestibility, improved in vitro ruminal fermentation parameters and decreased ruminal methane production. However, not all in vitro studies have reported reductions in methane production [10].

It has been hypothesized that LAB silage inoculants could reduce methane emissions from ruminants by several modes of action such as changes in the chemical composition of the silage, interaction of LAB with rumen microbes and alteration of rumen fermentation [11]. Methane produced from anaerobic fermentation in the rumen is the second most prevalent greenhouse gas with a global warming potential 23 times higher than that of carbon dioxide [12]. Therefore, reducing ruminal methane production not only improves the efficiency of nutrient utilization in ruminants but also helps to protect the environment from the negative consequences of global warming.

From the microbiological perspective, some studies indicated that the inclusion of silage alone [13], as well as silage in combination with LAB inoculant [4], could improve the microbial population in the rumen. However, to the best of our knowledge, there is still limited information on the effect of different types of LAB-inoculated rice straw silage on microbial population responses. Therefore, the purpose of this study was to test the rumen microbial populations and fermentation characteristics, as well as testing the methane mitigation potential of rice straw silage inoculated with different types of LAB in an in vitro condition.

## 2. Materials and Methods

### 2.1. Isolation, Identification and Characterization of LAB

Cecal contents obtained from healthy, adult, commercial broiler chickens at slaughterhouses, and rumen samples from three fistulated bulls (body weight: 210 kg) were used as sources for isolation of LAB. One gram of each sample was dissolved in 9 mL of peptone water (0.01%) and shaken at 200 rpm for 10 min. Several dilutions from each sample (10^−3^ to 10^−7^) were prepared in a dilution tube containing peptone water (0.01%). Then, 100 µL of each diluted sample were transferred onto a plate containing MRS Rogosa agar (Oxoid CM 627, Hampshire, UK) as a selective medium for LAB [14]. Plates were anaerobically incubated at 37 °C for 48 h. Several bacterial colonies were selected from each plate and subcultured three times. A total of 80 isolates were selected and tested for Gram stain, hydrogen peroxidase and lactic acid production. The LAB strains that actively produced lactic acid were chosen for the molecular identification.

### 2.2. Molecular Identification

DNA of selected LAB was extracted using a DNA extraction kit (QIAamp Blood and Tissue Kit, Qiagen, Hilden, Germany). The amplification of the 16SrRNA genes was conducted using 27F 5′-AGAGTTTGATCCTGGCTCAG-3′ and 1492R 5′-GGCTACCTTGTTACGACTT-3′primers. PCR amplification was performed with i-StarTaq DNA polymerase kit (iNtRON Biotechnology, Sungnam, Kyungki-Do, Korea) using 1 μL of a template (10 ng/μL) in 20 μL of the reaction solution. Amplification was performed using a BIORAD MyCycler™ thermal cycler with the following program: 1 cycle at 94 °C for 4 min, 30 cycles of 94 °C for 1 min, 55 °C for 30 s, 72 °C for 2 min and a final extension at 72°C for 5 min. The PCR products were mixed with loading dye and loaded on to a 1.0% SeaKem^®^ GTG^®^ agarose (FMC BioProducts, Rockland, ME, USA) containing ethidium bromide, and electrophoresis was carried out at 90 V for 1 h. The PCR products were visualized under UV illumination, excised from the gel, and the PCR product was extracted using MEGA quick-spin PCR & Agarose Gel Extraction kit (iNtRON Biotechnology). The PCR product was sequenced using forward and reverse primers (1st Base Co., Malaysia). The amplified products were analyzed by Sanger sequencing using the ABI 3730XL DNA analyzer (Applied Biosystems, Foster City, CA, USA). The contig was done for the forward and reverse sequences of each isolate by the contig assembly program of Bioedit v. 7.2.0 software [15] and then sequences were analyzed by the Bellerophon [16] and Mallard program [17] to remove chimeric rDNA clones. Approximately 1400 bp segments of the 16S rRNA gene of the isolates were subjected to analysis by BLAST using National Center for Biotechnology Information (NCBI) library with the following address: http://blast.ncbi.nlm.nih.gov/Blast.cgi, 21 February 2020. The isolates were identified according to the BLAST results and the 16S rRNA gene sequences submitted to NCBI GenBank. The obtained accession number were as follow: *L. plantarum* (KJ160209), *L. salivarius* (KJ160204), *L. reuteri* (KJ160196), *L. brevis* (KJ160214), *S. bovis* (KJ160185). All identified lactic acid bacteria were from the cecum of broiler chickens except for *S. bovis,* which was isolated from rumen samples.

### 2.3. Silage Preparation and Fermentation

Fresh rice straw was obtained from the fields of the Malaysian Agricultural Research and Development Institute (MARDI) located in Serdang, Selangor, Malaysia. Straw was chopped to 8–10 cm long pieces with a laboratory chopper. Five isolates of LAB (*L. plantarum, L. salivarius, L. reuteri, L. brevis and S. bovis*) were used for inoculation, and the inoculation rate was based on the numbers of colony-forming units per gram in the inoculant powders. The dry matter of chopped rice straw was determined, and the inoculants were applied by suspending the appropriate weight of inoculant powder in the required amount of water to increase the moisture content of rice straw to 70% and spraying it over 2 kg batches of rice straw and mixed thoroughly. Each treatment contained 10^6^ cfu/g DM of LAB inoculants. The treated rice straw was ensiled in 500 mL Schott bottles. There were three bottles per inoculant treatment of each of the silages. The silages were stored for 15 and 30 days at room temperature (28 to 32 °C). Control silages were also prepared at the same time with sterile water.

### 2.4. Chemical Analyses and Fermentation Quality for Rice Straw Silage

After 15 and 30 days of ensiling, bottles of the untreated and inoculated silages were opened for analyzing chemical composition and fermentation quality. The dry matter (DM), crude protein (CP) (total nitrogen × 6.25) and gross energy (GE) were determined using AOAC procedures [18]. Dry matter (DM) was determined by drying 10 g of fresh samples at 60 °C in a forced air oven for 48 h. The GE determined by using bomb calorimeter. Neutral detergent fiber (NDF) and acid detergent fiber (ADF) were determined according to Van Soest and coworkers [19]. Representative silage (20 g) was mixed with 180 g of sterile water in a laboratory blender (Waring, New Hartford, CT, USA) for 2 min. The extract was filtered through four layers of gauze and no. 1 filter paper (Whatman, Inc., Clifton, NJ, USA). The filtrate extract was used for analysis of NH_3_-N, pH, LAB population, lactic acid, volatile fatty acids (VFAs) and monosaccharides. The concentration of NH_3_-N was determined as described in our previous work [20]. The pH was determined from the filtrate solution using a pH electrode (Mettler-Toledo Ltd., Leicester, UK). The lactic acid and volatile fatty acids were determined using gas-liquid chromatography as described in our previous work, and the result was expressed as g/Kg DM of rice straw silage [21]. The total number of LAB in the silage was determined on MRS Rogosa agar, as described above, with the plate count method [14]. Colonies were counted from the plates at appropriate dilutions and the number of colony-forming units (cfu) was expressed as log10 per gram of fresh rice straw. The concentration of monosaccharides was determined by using HPLC (Waters 2690, Milford, MA, USA), using a COSMOSIL Sugar-D column (4.6 mm I.D. 250 mm) (Nacalai, San Diego, CA, USA). The solvent was acetonitrile/water (80:20; v/v) and the flow rate was 0.7 mL/min. Monosaccharides standards, including those for fructose, xylose and glucose, were purchased from Sigma-Aldrich (Sigma-Aldrich, St. Louis, MI, USA) [22].

### 2.5. In Vitro Rumen Fermentation and Digestibility

Three rumen-fistulated bulls (Kedah-Kelantan breed, *Bos indicus*) with body weight of about 210 kg were used as rumen fluid donors. Bulls were kept in a tie-stall housing system. The bulls were kept in stalls with a length of 250 cm, and width of 190 cm; bedding (straw) was used in all of the barns. Manure cleaning of the barns was made manually at the farm. Bulls were fed twice a day with mixed forage and concentrate (~60:40 ratios) at the maintenance level. The animals had free access to water and mineral blocks, and they had enough space to move. Table 1 shows the ingredients and chemical composition of the diets fed to the bulls for the in vitro study. Bulls were also drenched against parasites before the onset of the experiment. Rumen fluid was collected before the morning feeding from both fistulated bulls and strained through four layers of muslin gauze into a pre-warmed bottle at 39 °C. A total of six calibrated glass syringes (Haberle Labortechnik, Lonsee, Germany) for each treatment (each micro silage) were used for the in vitro study [23]. From six syringes for each treatment (each micro silage) the contents of three syringes were used for in vitro dry matter digestibility (IVDMD) and fermentation parameters, and the remaining three syringes were used for rumen microbial population quantification. Substrate (200 mg) was weighed into 100 mL calibrated glass syringes. The incubation medium was prepared as described previously [21,24], and 40 mL was dispensed anaerobically into each syringe. Syringes were incubated at 39 °C for 24 h. In vitro gas production was measured at 2, 4, 8, 12 and 24 h postincubation using three syringes/treatment for each incubation time. In each incubation run, three blanks without inoculum were used to correct the values for gas released from the substrates. The data for cumulative gas production were fitted to the model of Ørskov and McDonald [25] and the values of *a* (gas produced from quick soluble fraction), *b* (gas produced from the insoluble fraction), *a + b* (potential extent of gas production) and *c* (the rate of gas production from insoluble fraction) were estimated using the nonlinear regression (NLIN) procedure of SAS [24,26,27]. The above procedures were conducted in three individual runs. After 24 h of fermentation, the IVDMD of substrates was determined by the contents of syringes. Briefly, at the end of the 24 h incubation, the content of the gas syringes for all treatments, including the blank, were transferred into beakers predried in a 100 °C oven overnight, with the dry weight of each beaker being recorded and labeled accordingly. Distilled water was used to rinse the interior and the plunger of the syringes to reduce the chances of overestimation of IVDMD. The beakers were then incubated at 100 °C until their contents dried up and their weight had become stable before weight determination. The fermentation end products (e.g., pH, NH_3_-N and VFAs) and the number of LAB were also determined as described earlier [24]. The protocol for the experimental procedures were reviewed and approved by the Animal Care and Use Committee of the University of Putra in Malaysia.

### 2.6. Quantification of Rumen Microbial Population by Real-Time PCR

The quantification of targeted microbes, including cellulolytic bacteria such as *Fibrobacter succinogenes, Ruminococcus albus, Ruminococcus flavefaciens,* general bacteria, general anaerobic fungi, total protozoa, total methanogens and total archaea, was conducted as described in detail in our previous studies [24,27]. Briefly, the DNA was extracted from 300 µL of fermented rumen content (fluid and digesta) by QIAGEN DNA Mini Stool Kit (QIAGEN, Valencia, CA, USA) according to the manufacturer’s recommendations. The 16S rRNA of bacteria and 18S rRNA of protozoa and fungi were amplified by PCR (Bio-Rad, Hercules, CA, USA). The primer sets used in the current study are shown in Table 2. The PCR product was purified using a QIA quick PCR purification kit (QIAGEN, Inc., Valencia, CA, USA) and cloned in pCR^®^2.1-TOPO^®^ TA cloning vector (Invitrogen, Carlsbad, CA, USA) and transformed into chemically competent *E. coli* TOP10 cells (Invitrogen, Carlsbad, CA, USA).The plasmid was extracted from all white colonies (ranged from 9–24 colonies). The extracted plasmids (ranged from 9–24 plasmids) were analyzed by EcoR1 restriction enzyme and separated on a 1% (*w*/*v*) agarose gel to confirm the presence and correct orientation of the inserts. Plasmids with the right cloned inserts were sequenced using an Applied Biosystems 3730xl DNA analyzer (Applied Biosystems, Foster City, CA, USA) in order to assert their identity. Bellerophon software was used to check the chimeric rDNA [16], and the sequences were blast-identified using basic alignment tool with those available in the GenBank [28]. Plasmids possessing sequences with more than 99% similarity to previously published sequences of target microorganisms were applied for standard curve construction by real-time PCR. The concentration and purity of the plasmids were evaluated using Nanodrop (Nanodrop Technologies, Wilmington, DE, USA) and the copy number was determined using below formula [29].

$$\frac{Amount\;of\;DNA\;\left(µg/\mathrm{mL}\right)\times 6.022\times 10^{23}}{Length\;\left(\mathrm{bp}\right)\times 10^{9\;}\times 650}$$

A Bio-Rad CFX 96 real-time PCR thermocycler (Bio-Rad, Hercules, CA, USA) and SYBR Green (iQ Supermix, Bio-Rad Laboratories, Inc., Hercules, CA, USA) were used in this study. All the amplifications were conducted in triplicate and the data obtained from real-time PCR amplification were analyzed using CFX manager version 3 (Bio-Rad Laboratories, Alfred Nobel Drive, Hercules, CA, USA).

### 2.7. Statistical Analyses

The general linear model (GLM) procedure of SAS in a completely randomized design (CRD) was used for the statistical analysis following the model: Yi = μ Ti+ei, where μ is the value of the mean, Ti is the effect of treatment and ei is the error of experiment, respectively. Means were compared with Duncan’s Multiple Range test and considered significantly different at *p* < 0.05 [26].

## 3. Results

### 3.1. Chemical Analyses and Fermentation Quality of Rice Straw Silage

The contents of DM, CP, ether extract, NDF and ADF were affected (*p* < 0.05) by the treatments (Table 3). The DM contents were decreased as the duration of ensiling increased. The CP content was greater in LAB treatments (*L. plantarum, L. salivarius, and S. bovis*) as compared with control (*p* < 0.05). The NDF and ADF of the LAB treatments were less than those of the control (Table 3). However, the gross energy was not affected (*p* > 0.05) by the treatments. Analysis of sugar in fermented rice straw showed a significant decrease (*p* < 0.05) in the concentration of glucose and fructose among LAB treatments as compared with the control at 30 days of incubation.

The value of pH decreased in all treatments except for control as the duration of ensiling increased (from 15 d to 30 d). The LAB treatment groups showed the lowest pH value as compared with control throughout the ensiling period, with pH values between 4.3–5.3. Lactic acid content (g/Kg DM) increased from days 15 to 30 among all treatments; however, LAB treatments were significantly higher than that of control. Among the LAB treatments, *L. plantarum* and *S. bovis* had the highest lactic acid content at 30 d of ensilage (36.9 and 35.7, g/Kg DM, respectively). The acetic acid and propionic acid contents of all treatments increased with the increase in duration of ensiling. Again, *L. plantarum* and *S. bovis* showed the greatest values for acetic and propionic acids at 30 d of ensilage (24.1 and 2.9 vs. 22.5 and 2.5 g/Kg DM, respectively). Butyric acid content showed a decreasing trend among the treatments as the duration of ensiling increased, with the highest value for the control (5.5 and 4.6 g/Kg DM at 15 and 30 d of ensilage, respectively). Compared with the control, LAB treatments did not show significant differences (*p* > 0.05) in terms of NH_3_-N concentration (average: 0.049%). The analysis of the LAB content (log cfu/g) showed that the LAB treatments exhibited a significant (*p* < 0.05) increase as compared to that of control (Table 4).

### 3.2. In Vitro Rumen Fermentation Characteristics, Methane Production and DM Digestibility

According to the in vitro data (Table 5), LAB treatments had less (*p* < 0.05) gas production at 24 h of fermentation compared with control. Conversely, the coefficient of degradable *B* fraction was greater in LAB treatments, especially for *L. plantarum* and *S. bovis* (at 30 d of ensilage) compared with the control. However, coefficients of rapidly degradable *a* fraction and *c* (degradation rate of degradable b fraction) were not affected (*p* > 0.05) by the treatments. The LAB treatments, especially for *L. plantarum* and *S. bovis* at 30 d of ensiling, had greater (*p* < 0.05) amounts of IVDMD as compared with control. Total VFA and acetic acid were also greater (*p* < 0.05) among LAB treatments. The concentration of NH_3_-N and pH were similar among the treatments (*p* > 0.05). Methane production and methane/total gas significantly (*p* < 0.05) decreased between LAB treatments compared with control groups. The *L. plantarum* at 15 and 30 d ensiling exhibited, respectively, 46% and 48% of CH_4_ reduction as compared to the control.

### 3.3. In Vitro Rumen Microbial Populations

The LAB treatments had greater (*p* < 0.05) total bacteria and fungi at 24 h of fermentation as compared with controls (Table 6). Conversely, controls had greater (*p* < 0.05) total protozoa, methanogens, and archaea at the end of in vitro fermentation. Butyrivibrio fibrisolvens and Ruminococcus flavefaciens was lower for controls compared with LAB treatments at 15 and 30 days of silage. Especially, *L. plantarum* and *S. bovis* at 30 d of silage had the greatest populations of *B. fibrisolvens* and *R. flavefaciens*. *Fibrobacter succinogenes* were also almost similar among LAB treatments but higher (*p* < 0.05) than the control group. Overall, *L. plantarum, L. brevis*, and *S. bovis* showed more similar characteristics as compared with others in terms of microbial populations.

## 4. Discussion

### 4.1. Chemical Composition and Fermentation Characteristics

Inoculating the different types of LAB for ensiled rice straw increased CP content in the current study. This increase in the CP content might be partially attributed to microbial growth. Our results were in line with the results of [6] in which LAB culture broth affected the feed quality of rice straw; however, our results showed no effect on NH_3_-N concentration, which was contradictory to theirs. High concentration of NH_3_-N could probably be attributed to excessive breakdown of protein during fermentation, which lowers silage quality [12]. Lower NDF and ADF contents among the LAB treatments compared with the control group could be the result of the lower level of heat damage of the protein, which improves energy content [30] as shown in Table 3. A study [31] mentioned that lower NDF content in silage could also be due to the loss of hemicellulose occurring in the ensiling process. This loss could be due to a combination of enzymatic and acid hydrolysis of the more digestible cell wall fractions during fermentation. Ensiling of the forage mostly results in DM loss, which occurs during fermentation. The lack of DM loss in our study was also reported with the application of LAB isolated from forage paddy rice silage in China [32].

Previous studies showed that bacterial inoculation of silage could convert the composition of cell-wall carbohydrates into organic acids and cause a decrease in pH during fermentation [32]. The decrease in the pH could inhibit *Clostridium* spp. and aerobic bacteria growth [32]. In the current study, pH decreased with increases in the duration of ensiling among the LAB treatments (especially for *L. plantarum* and *S. bovis*) which agreed with those in the literature. Silage pH (the lower the better) is one of the main factors depicting the extent of fermentation and quality of ensiled forage [31]. The lower pH among LAB treatments (4.99) and VS control (5.6) suggests good fermentation. Similarly, Kim et al. [25] indicated that *L. plantarum* inoculant for fresh rice straw silage decreased pH, as well as acetic acid, NH_3_-N and butyric acid contents [33]. However, in our study, LAB treatments increased acetic acid with no effect on butyric acid content. A high concentration of butyric acid could be a sign of protein degradation and DM loss, as well as energy waste [7]. Kim et al. [25] also concluded that adding *L. plantarum* could improve the fermentation quality and feed value of rice straw silage. Inoculation of the mixture of corn steep liquor and air-dried rice straw with homofermentative (*L. plantarum*) and heterofermentative (*L. plantarum, Lactobacillus casei,* and *Lactobacillus buchneri*) LAB significantly increased the concentration of acetic acid and lactic acid compared with the control in a study conducted in China [34]. Our results are consistent with that study in terms of increased acetic acid and lactic acid contents. The high concentration of lactic acid results in lower pH (as observed in this study) which inhibits the growth and activities of undesirable bacteria during ensiling [7]. Acetic acid also possesses antifungal activity, which reduces the spoilage of organisms in the mass and improves the fermentation quality of silage. Zhang et al. [8] reported that chopping rice straw before ensiling could enhance the lactic acid concentration and total VFAs content. A study [34] also demonstrated that homo fermentative and heterofermentative LAB could effectively improve the fermentation quality of the silage. Rice straw, a by-product of rice production can be abundantly found in Southeast Asia, which is the most important rice-producing region in the world [8]. Thus, by improving the nutritive value of rice straw through fermentation processes, farmers could increase the application of rice straw as an animal feed and, therefore, overcome the limitations of feed sources in many parts of the tropics.

### 4.2. In Vitro Rumen Fermentation Characteristics, Methane Production and DM Digestibility

Some studies have reported the effectiveness of LAB inoculation on in vitro ruminal fermentation characteristics [1,35,36]. Lack of effect on rumen pH and NH_3_-N after 15 and 30 days of ensiling among the LAB treatments in this study was contrary to the results of [8]. They reported that three levels of LAB inoculants (LAB; 2×10^5^, 3×10^5^ and 4×10^5^ cfu/g fresh forage) on rice straw (whole and chopped rice straw) silage decreased pH, as well as NH_3_-N and acetic acid concentrations in Holstein dairy cows in an in vivo study. Our results are consistent with theirs in terms of total VFA and propionic acid concentrations, which showed respectively increase and decrease among the LAB treatments. A study [8] also concluded that the chopping process and LAB addition improved the silage quality of rice straw, and its partial substitution with corn silage could lower the cost of the dairy cow ration with no negative effects on lactation performance. Supplementing rice straw and sugar beet leaf silage treated with lactic acid bacteria enhanced the performance and productivity of lactating Friesian cows in an in vivo study [37]. Another in vivo study showed improved fermentation quality, as well as improved digestibility of feed components, after feeding wethers with urea-treated rice straw silage with LAB [38]. In this study, the LAB treatments, especially for *L. plantarum* and *S.bovis*, showed the highest IVDMD and the lowest methane production. Different results obtained among variant types of LAB in this study were consistent with [11], who showed that organic matter digestibility, gas, and methane production varied with the type of LAB added and type of substrate incubated. The higher IVDMD in *L. plantarum* and *S.bovis* compared with other LAB treatments could be the results of better utilization of the water-soluble carbohydrates, higher production of lactic acid and rapid decrease in pH [39]. A study [31] also mentioned that *L. plantarum* is the most commonly used silage inoculant. Our results confirm the previous studies and the results of [9] in which vegetable residue silage inoculated with *L. plantarum* showed the highest IVDMD and lowest methane production. Methane is a by-product of anaerobic fermentation of dietary carbohydrates in the rumen, and methanogenesis possesses a biological regulatory mechanism for animal health [40]. However, A study [20] mentioned that methane formation is a contributing factor to atmospheric burden of greenhouse gases, which is linked to global warming and climate change, as well as a significant energy loss for the animal due to the exit of carbon.

### 4.3. In Vitro Rumen Microbial Populations

Ruminal in vitro dry matter degradability is the result of microbial decomposition of nutrients, and increase in degradability is a good indicator of animal production performance, which is associated with well-fermented silages [41]. One of the potential alternatives for the replacement of antibiotics in livestock production is the use of direct-fed microbials as feed additives [42]. LAB, as a particular type of direct-fed microbial, as well as LAB silage inoculants, have exerted probiotic effects resulting in improvement in ruminant performance [43]. In the current study, microbial populations were affected by the LAB treatments. *Fibrobacter succinogenes*, *Ruminococcus flavefaciens* and *Ruminococcus albus,* which are the most predominant cellulolytic bacterial species in ruminants, were highest among the LAB treatments. The increase in the population of these major cellulolytic bacteria is attributed to the increase in the dry matter degradability, or fermentation metabolites, produced by lactic acid bacteria during fermentation of rice straw. Similar to the observation made in this experiment, [13] reported that dairy steers receiving rice straw and *Leucaena* silage enhanced rumen microbial population (especially cellulolytic), and fungal population as well. They mentioned a decrease in the protozoal populations by the increase in the level of *Leucaena* silage. We also found decreases in the protozoal and methanogen populations by the LAB treatments. A study [12] reported that protozoa can provide electrons through the activity of hydrogenases and, hence, antiprotozoal effects of feedstuff could decrease methane production by methanogens attached to protozoa.

LAB, or their metabolites, may affect the methanogens themselves, or they may affect t other rumen microbes that produce H_2_ or methyl-containing compounds, which are the substrates for methanogenesis [44]. LAB and their metabolites, such as bacteriocins, may directly inhibit rumen methanogens, protozoa and even archaea, resulting in reduction of methane production. The results of Callaway, et al. [45] indicted the potential application of nisin, a bacteriocin from *Lactococcus lactis,* in methane reduction in in vitro rumen fermentation by 36%. Another study reported a 10% decrease in the methane production in an in vivo trial by feeding bacteriocin to sheep [46]. A further study conducted by Astuti, et al. [47] revealed that *L. plantarum* could stimulate the growth of lactate-utilizing bacteria, leading to increased production of propionic acid and a subsequent decrease in the hydrogen availability for methane production. The results of Cao, et al. [48,49], are in agreement with our observation which the silage fermentation by LAB improves in vitro dry matter digestibility and lowers methane production.

Moreover, fungal populations were increased in our studies among the LAB treatment groups. A study [13] indicated that there was an increase in the numbers of fungi when protozoa were removed from the rumen. They also mentioned that *Leucaena* silage could provide an adequate nitrogen source for microbial growth, leading to increase in the bacterial population, which could be the case for our results. Consistent with our study, total mixed rations containing corn silage and/or grass silage increased total bacteria and *Fibrobacter succinogenes* in dairy cows [50]. *B. fibrisolvens* which is involved in rumen fatty acid biohydrogenation, was greater among LAB treatments in this study. Conjugated linoleic acid, which has beneficial biological effects in animal models, is formed as an intermediate during biohydrogenation of linoleic acid to stearic acid in the rumen by mainly *B. fibrisolvens* and other rumen bacteria [51].

## 5. Conclusions

In conclusion, inoculation of lactobacilli (10^6^ cfu /g DM) in rice straw silage improved silage quality (e.g., high CP content) and fermentation characteristics (e.g., increase in production of lactic acid and acetic acid). Among inoculated LAB, *L. plantarum* and *S. bovis* were found to be more potent for fermentation. An in vitro rumen digestibility test showed higher rumen digestibility, higher VFA production and lower methane production in the rice straw fermented with LAB, particularly with *L. plantarum* and *S. bovis.* Moreover, analysis of the rumen microbial population showed significant increases in the populations of cellulolytic bacterial (*Fibrobacter succinogenes*, *Butyrivibrio fibrisolvens* and *Ruminococcus flavefaciens*), protozoa, methanogens and archaea among the LAB treatments compared with controls. Overall, *L. plantarum, S. bovis* were found to be more promising to be applied in rice straw fermentation; however, in vivo experiments need to confirm these results.

## Figures and Tables

**Table 1:** Ingredients and chemical composition of the diets fed to the bulls for in vitro study.

Ingredients (/kg DM)	g/kg DM
Alfalfa Hay	314.1
Corn, grain	170.0
Soybean meal	133.0
Palm kernel cake	251.1
Rice bran	81.8
Sunflower oil	20.0
Mineral premix	5.0
Vitamin premix	5.0
Ammonium chloride	10.0
Limestone	10.0
Chemical composition	g/kg DM
DM	850.2
CP	208.3
EE	52.5
NDF	419.0
ADF	253.0

DM, dry matter; CP, crude protein, EE, ether extract; NDF, neutral detergent fiber; ADF, acid detergent fiber.

**Table 2:** Microorganisms and characteristics of the primers used in this study [24,27].

Target Microorganism	Primer Sequences (5′–3′)
*Fibrobacter succinogenes* F	GGTATGGGATGAGCTTGC
*Fibrobacter succinogenes* R	GCCTGCCCCTGAACTATC
*Ruminococcus albus* F	CCCTAAAAGCAGTCTTAGTTCG
*Ruminococcus albus* R	CCTCCTTGCGGTTAGAACA
*Ruminococcus flavefaciens* F	CGAACGGAGATAATTTGAGTTTACTTAGG
*Ruminococcus flavefaciens* R	CGGTCTCTGTATGTTATGAGGTATTACC
General bacteria F	CGGCAACGAGCGCAACCC
General bacteria R	CCATTGTAGCACGTGTGTAGCC
General anaerobic fungi F	GAGGAAGTAAAAGTCGTAACAAGGTTTC
General anaerobic fungi R	CAAATTCACAAAGGGTAGGATGATT
Total protozoa F	GCTTTCGWTGGTAGTGTATT
Total protozoa R	CTTGCCCTCYAATCGTWCT
Total methanogens F	GCTCAGTAACACGTGG
Total methanogens R	CGGTGTGTGCAAGGAG
Total archaea F	ATTAGATACCCSBGTAGTCC
Total archaea R	GCCATGCACCWCCTCT

F: forward; R: reverse.

**Table 3:** Effect of inoculation of LAB on the chemical composition of ensiled rice straw (DM basis).

Treatments	Day	DM (%)	CP (%)	NDF (%)	ADF (%)	GE (Kcal)	Glucose ^1^	Fructose ^1^	Xylose ^1^
Control	15	34.8 ^a^	9.3 ^c^	69.2 ^a^	56.2 ^a^	15.7	5.3 ^a^	0.4 ^a^	0.4
	30	33.6 ^a^	9.8 ^c^	70.2 ^a^	56.8 ^a^	15.9	4.8 ^b^	0.4 ^a^	0.4
*L. plantarum*	15	30.5 ^bc^	11.8 ^b^	66.8 ^c^	53.3 ^b^	15.5	3.5 ^c^	0.3 ^ab^	0.4
	30	29.2 ^c^	12.7 ^a^	64.1 ^d^	49.4 ^c^	14.8	1.2 ^h^	0.1 ^c^	0.3
*L. salivarius*	15	33.2 ^a^	11.2 ^b^	68.9 ^a^	53.8 ^b^	15.7	3.6 ^c^	0.3 ^ab^	0.4
	30	32.8 ^ab^	12.1 ^ab^	66.6 ^c^	51.9 ^c^	15.5	2.5 ^f^	0.2 ^b^	0.4
*L. reuteri*	15	32.5 ^ab^	10.9 ^bc^	67.6 ^b^	53.7 ^b^	15.9	3.2 ^d^	0.3 ^ab^	0.4
	30	31.3 ^b^	10.9 ^bc^	66.6 ^c^	52.5 ^bc^	15.0	2.4 ^f^	0.2 ^b^	0.3
*L. brevis*	15	33.4 ^a^	10.6 ^bc^	66.9 ^c^	52.9 ^bc^	15.3	3.3 ^d^	0.3 ^ab^	0.4
	30	32.4 ^ab^	10.9 ^bc^	66.6 ^c^	50.6 ^c^	15.1	3.0 ^e^	0.2 ^b^	0.3
*S. bovis*	15	32.6 ^ab^	12.4 ^a^	67.5 ^b^	52.1 ^bc^	15.7	3.4 ^cd^	0.3 ^ab^	0.3
	30	31.5 ^b^	12.5 ^a^	64.8 ^d^	50.8 ^c^	15.3	1.9 ^g^	0.2 ^b^	0.3
SEM ^2^	-	0.60	0.43	0.46	0.97	0.65	0.83	0.05	0.08

^1^ Unit: mg/g DM. DM (%), dry matter; CP (%), crude protein; NDF (%), neutral detergent fiber; ADF (%), acid detergent fiber; GE (kcal), gross energy. ^abcdefg^ Means in each column with different superscripts are significantly different (*p* < 0.05). ^2^ SEM, standard error of the mean.

**Table 4:** Effects of inoculation of LAB on fermentation quality of ensiled rice straw.

Treatments	Day	pH	Lactic Acid (g/Kg DM)	Acetic Acid (g/Kg DM)	Propionic Acid (g/Kg DM)	Butyric Acid (g/Kg DM)	NH_3_-N (%)	LAB (log cfu/g)
Control	15	5.6 ^a^	5.1 ^f^	10.5 ^e^	1.3 ^b^	5.5 ^a^	0.05	5.2 ^c^
	30	5.6 ^a^	9.4 ^e^	11.6 ^e^	1.6 ^b^	4.6 ^a^	0.05	5.8 ^c^
*L. plantarum*	15	5.2 ^ab^	19.6 ^c^	20.8 ^b^	1.9 ^ab^	1.7 ^cd^	0.04	6.6 ^bc^
	30	4.4 ^b^	36.9 ^a^	24.1 ^a^	2.9 ^a^	1.7 ^cd^	0.05	8.8 ^a^
*L. salivarius*	15	5.4 ^a^	14.6 ^d^	13.4 ^d^	1.2 ^b^	3.3 ^b^	0.05	6.5 ^bc^
	30	4.8 ^b^	22.4 ^bc^	19.1 ^bc^	1.3 ^b^	2.4 ^b^	0.05	7.3 ^b^
*L. reuteri*	15	5.5 ^a^	15.5 ^d^	14.2 ^d^	1.5 ^b^	2.2 ^c^	0.05	6.4 ^bc^
	30	4.8 ^b^	26.6 ^b^	17.4 ^c^	2.1 ^ab^	2.1 ^c^	0.06	7.1 ^b^
*L. brevis*	15	5.3 ^a^	16.9 ^c^	14.4 ^d^	1.4 ^b^	2.6 ^b^	0.05	6.6 ^bc^
	30	4.9 ^b^	24.1 ^b^	18.1 ^c^	1.7 ^b^	2.1 ^c^	0.05	7.3 ^b^
*S. bovis*	15	5.3 ^a^	19.9 ^c^	19.5 ^bc^	1.6 ^b^	1.7 ^cd^	0.04	6.8 ^bc^
	30	4.3 ^b^	35.7 ^a^	22.5 ^ab^	2.5 ^a^	1.2 ^d^	0.05	8.2 ^ab^
SEM ^1^	-	0.31	1.14	0.88	0.53	1.01	0.004	0.48

^abcdef^ Means in each column with different superscripts are significantly different (*p* < 0.05). ^1^ SEM, standard error of the mean.

**Table 5:** Effects of inoculation of LAB on in vitro rumen fermentation.

Treatments	Day	DMD	Total Gas ^1^	pH	NH_3-_N	Total VFA (mM)	Acetic Acid	Propionic Acid	CH_4_	CH_4_/Total Gas
Control	15	21.4 ^f^	45.5 ^a^	6.9	14.3	65.6 ^g^	45.5 ^g^	13.6 ^a^	7.8 ^a^	0.17 ^a^
	30	22.2 ^f^	45.0 ^a^	6.9	15.6	67.5 ^ef^	44.5 ^g^	12.7 ^ab^	7.9 ^a^	0.18 ^a^
*L. plantarum*	15	25.4 ^de^	42.0 ^bc^	6.9	15.3	74.7 ^c^	46.1 ^fg^	12.4 ^b^	4.2 ^c^	0.10 ^cd^
	30	29.4 ^a^	37.5 ^c^	6.9	15.9	79.7 ^a^	54.4 ^a^	12.9 ^ab^	4.1 ^c^	0.11 ^cd^
*L. salivarius*	15	22.4 ^f^	43.5 ^b^	6.8	15.2	68.8 ^e^	47.0 ^f^	12.4 ^b^	6.1 ^b^	0.14 ^b^
	30	26.4 ^cd^	41.0 ^c^	6.8	15.8	76.1 ^b^	49.7 ^d^	13.0 ^ab^	5.9 ^b^	0.14 ^b^
*L. reuteri*	15	22.4 ^f^	40.5 ^c^	6.8	14.9	70.6 ^d^	46.7 ^f^	13.3 ^a^	4.5 ^c^	0.11 ^cd^
	30	26.4 ^cd^	40.0 ^cd^	6.8	15.5	74.5 ^c^	51.6 ^c^	13.2 ^a^	4.6 ^c^	0.12 ^c^
*L. brevis*	15	22.1 ^f^	43.0 ^b^	6.8	15.3	67.2 ^ef^	48.3 ^de^	12.5 ^b^	5.2 ^b^	0.12 ^c^
	30	27.4 ^bc^	42.0 ^bc^	6.8	15.6	78.3 ^ab^	52.7 ^b^	13.6 ^a^	5.4 ^b^	0.13 ^bc^
*S. bovis*	15	24.4 ^ef^	40.5 ^c^	6.8	15.1	74.5 ^c^	49.2 ^d^	13.8 ^a^	5.7 ^b^	0.14 ^b^
	30	28.4 ^ab^	39.5 ^cd^	6.8	15.7	78.9 ^a^	53.5 ^ab^	13.3 ^a^	5.2 ^b^	0.13 ^bc^
SEM ^2^	-	0.68	0.84	0.03	0.48	1.07	0.48	0.32	0.26	0.004

^1^ Total gas (mL/200 mg DM), NH_3_-N (mg/dL), a, b, c, and a + b were calculated from exponential equation *p* = a + b (1 − e^ct^). a = gas production from the immediately soluble fraction (%), b = gas production from the insoluble fraction (%), c = gas production rate constant for the insoluble fraction (b) (fraction/h), (a + b) = potential extent of gas production. DMD, dry matter digestibility; VFA, volatile fatty acid. ^2 abcdefg^ Means in each column with different superscripts are significantly different (*p* < 0.05).

**Table 6:** Effects of inoculation of LAB on in vitro rumen microbial populations.

Treatment ^1^	Day	*Fibrobacter succinogenes*	*Butyrivibrio fibrisolvens*	*Ruminococcus flavafaciences*	Total Bacteria	Total Fungi	Total Protozoa	Total Methanogens	Total Archaea
Control	15	1.54 ^b^	0.40 ^c^	1.20 ^c^	0.53 ^b^	0.76 ^b^	3.93 ^a^	7.62 ^a^	5.78 ^a^
	30	1.61 ^b^	0.72 ^c^	1.31 ^bc^	1.05 ^ab^	0.78 ^b^	3.99 ^a^	7.57 ^a^	5.78 ^a^
*L. plantarum*	15	1.82 ^ab^	1.28 ^b^	1.91 ^b^	1.42 ^a^	1.07 ^ab^	3.62 ^ab^	7.34 ^ab^	5.34 ^ab^
	30	2.50 ^a^	2.32 ^a^	2.68 ^a^	1.56 ^a^	1.37 ^a^	3.49 ^b^	7.11 ^b^	4.91 ^b^
*L. salivarius*	15	1.64 ^b^	1.12 ^c^	1.33 bc	1.36 ^ab^	0.83 ^b^	3.84 ^a^	7.41 ^a^	5.50 ^a^
	30	1.89 ^ab^	1.88 ^ab^	2.19 ^ab^	1.43 ^a^	1.15 ^a^	3.74 ^ab^	7.29 ^ab^	5.25 ^ab^
*L. reuteri*	15	1.64 ^b^	0.94 ^c^	1.31 ^bc^	1.33 ^ab^	0.83 ^b^	3.86 ^a^	7.47 ^a^	5.35 ^ab^
	30	1.89 ^ab^	1.35 ^b^	2.07 ^ab^	1.42 ^a^	1.12 ^a^	3.66 ^ab^	7.36 ^ab^	5.01 ^b^
*L. brevis*	15	1.71 ^b^	1.13 ^c^	1.35 ^bc^	1.39 ^ab^	0.96 ^ab^	3.79 ^ab^	7.51 ^a^	5.43 ^ab^
	30	2.12 ^a^	1.91 ^ab^	2.29 ^a^	1.44 ^a^	1.23 ^a^	3.66 ^ab^	7.43 ^a^	5.11 ^b^
*S. bovis*	15	1.77 ^b^	1.16 ^bc^	1.80 ^b^	1.42 ^a^	0.97 ^ab^	3.62 ^ab^	7.44 ^a^	5.22 ^ab^
	30	2.24 ^a^	2.13 ^a^	2.55 ^a^	1.47 ^a^	1.34 ^a^	3.53 ^b^	7.22 ^b^	4.99 ^b^
SEM ^2^	-	0.15	0.14	0.13	0.15	0.22	0.28	0.48	0.23

^1^*Fibrobacter succinogenes*, ×10^7^ copies/1 mL of rumen fluid & digesta; *Butyrivibrio fibrisolvens*, ×10^4^ copies/1 mL of rumen fluid & digesta; *Ruminococcus flavefaciens,* ×10^6^ copies/1 mL of rumen fluid & digesta; Total bacteria, ×10^10^ copies/1 mL of rumen fluid & digesta; Total fungi, ×10^7^ copies/1 mL of rumen fluid & digesta; Total protozoa, ×10^7^ copies/1 mL of rumen fluid & digesta; Total methanogens, ×10^7^ copies/1 mL of rumen fluid & digesta; Total archaea, ×10^8^ copies/1 mL of rumen fluid & digesta. ^abc^ Means in each column with different superscripts are significantly different (*p* < 0.05). ^2^ SEM, standard error of the mean.

## Data Availability

The datasets during and/or analyzed during the current study are available from the corresponding author on reasonable request.
